# Resting heart rate is an independent predictor of advanced colorectal adenoma recurrence

**DOI:** 10.1371/journal.pone.0193753

**Published:** 2018-03-02

**Authors:** Jihye Park, Jae Hyun Kim, Yehyun Park, Soo Jung Park, Jae Hee Cheon, Won Ho Kim, Ji Soo Park, Justin Y. Jeon, Tae Il Kim

**Affiliations:** 1 Department of Internal Medicine, Yonsei University College of Medicine, 50 Yonsei-ro, Seodaemun-gu, Seoul, Korea; 2 Institute of Gastroenterology, Yonsei University College of Medicine, 50 Yonsei-ro, Seodaemun-gu, Seoul, Korea; 3 Cancer Prevention Center, Yonsei University College of Medicine, 50 Yonsei-ro, Seodaemun-gu, Seoul, Korea; University Hospital Llandough, UNITED KINGDOM

## Abstract

**Background and aim:**

High heart rate is an independent predictor of total cancer incidence and all-cause mortality in patients with cancer. We aimed to evaluate the impact of resting heart rate on the recurrence of colorectal polyp, using long-term surveillance follow-up data of colorectal cancer survivors.

**Methods:**

Three hundred patients were selected from the colorectal cancer survivor cohort of Severance Hospital, Seoul, Korea. Resting heart rate, physical activity, and body composition analysis at the time of 5-year survival, and clinical data including colonoscopy surveillance results were collected for mean follow-up duration of 8 years.

**Results:**

Patients with a high resting heart rate showed a significantly higher recurrence rate of advanced adenoma than those with a low resting heart rate (quartile 1, 45–66 beats per minute (b.p.m.); quartile 2, 67–73 b.p.m.; quartile 3, 74–80 b.p.m.; quartile 4, 81–120 b.p.m.; 3.8% vs. 7.9% vs. 10.0% vs. 14.7%, *p* for trend = 0.018). After adjustment for various risk factors, patients in the highest quartile of resting heart rate (≥ 81 b.p.m.) had a significantly higher risk of advanced adenoma recurrence (hazard ratio [HR]: 6.183, 95% confidence interval [CI]: 1.181–32.373, *p* = 0.031), compared to those in the lowest quartile (≤ 66 b.p.m.). In subgroup analysis, the association of resting heart rate with advanced adenoma recurrence appeared to be stronger among patients who had more than normal body fat mass or sedentary life style.

**Conclusions:**

Elevated resting heart rate was independently associated with a higher rate of advanced adenoma recurrence in colorectal cancer survivors.

## Introduction

Resting heart rate is a sensitive indicator of the autonomic nervous system. An increase in the resting heart rate is caused by activation of sympathetic activity more than parasympathetic activity and/or decreased vagal tone. Resting heart rate can be influenced by lifestyle factors, such as psychological stress and physical activity [1,2]. Knowing that psychological stress and physical activity are closely associated with prognosis of cancer, high resting heart rate may be used as a prognostic factor for cancer recurrence. In a recent meta-analysis, the authors found an increased risk of coronary heart disease, sudden cardiac death, heart failure, atrial fibrillation, stroke, cardiovascular disease, total cancer, and all-cause mortality in patients with a higher resting heart rate [3–5]. The relative risk (RR) of a 10-beats-per-minute (b.p.m.) increase in the resting heart rate for patients with cancer was 1.14 (95% confidence interval [CI]: 1.06–1.23, *p* < 0.0001), and the RR of a high versus low resting heart rate was 1.43 (95% CI: 1.12–1.82, *p* < 0.0001) [3].

Colorectal cancer (CRC) is highly prevalent, with its incidence increasing worldwide [6,7]. Several factors, such as obesity, physical inactivity, Western diet, smoking, alcohol use, and a personal history of CRC or polyps, have been linked to CRC development and recurrence [8,9]. Interestingly, in a previous study, a resting heart rate ≥ 75 b.p.m. was an independent predictor of death in patients with colorectal, pancreatic, and non-small cell lung cancer (hazard ratio [HR]: 1.67, 95% CI: 1.01–2.78, *p* = 0.040) [10]. Another study showed a relationship between an elevated resting heart rate and an incident CRC risk in patients with manifest vascular disease (HR: 1.19, 95% CI: 1.00–1.42) [11].

The mechanisms underlying the relationship between resting heart rate and cancer are complex and not well understood. An increased heart rate impairs endothelial function in animal models and may contribute to reduced shear stress and vascular compliance [1,12]. Non-cardiac conditions, such as chronic inflammation associated with microvascular disease, might also be on the result of a high heart rate [13]. Moreover, increased sympathetic activation might also contribute to the initiation and progression of cancer, including inflammation, angiogenesis, tissue invasion, cellular immune response, and epithelial-mesenchymal transition [14].

To our knowledge, there has been no study that evaluated the association of heart rate with polyp recurrence in detail for a long-term surveillance period. In this study, we aimed to evaluate the impact of resting heart rate on the recurrence of colorectal polyp, using long-term surveillance follow-up data of CRC survivors.

## Patients and methods

### Patients

A total of 300 patients were randomly selected from the CRC survivor cohort of Severance Hospital, Seoul, Korea. These patients were followed-up for > 5 years after the diagnosis and treatment of CRC. The mean follow-up period from firstly diagnosed and treated was 8.0 years (median, 7 years; range, 5–21 years). After the curative resection of CRC, all patients underwent surveillance colonoscopy by experienced endoscopists. All patients underwent a baseline colonoscopy before colorectal resection or within 6 months after colorectal resection. In cases of incomplete colonoscopy before surgery, additional colonoscopic examinations were carried out within 6 months after the surgery, and the findings were included as part of the baseline colonoscopic findings. We excised all polyps detected during preoperative and postoperative colonoscopies, and all specimens were sent to the pathology department for histological evaluation. Follow-up colonoscopy was performed 1–4 times per patient, based on the guidelines [15]. Moreover, to overcome the interval diversities in surveillance colonoscopy, we investigated the total follow-up period and the timing of follow-up colonoscopy. The exclusion criteria were as follows: (i) incomplete medical records, (ii) a history of familial polyposis syndrome or Lynch syndrome, (iii) known inflammatory bowel disease (IBD), and (iv) incomplete baseline colonoscopy before colorectal resection or within 6 months after surgery. Cases of incomplete colonoscopy (nonvisualized cecum and/or inadequate bowel preparation) were also excluded.

Patients were identified using the electronic medical record system that includes all patients with cancer at Severance Hospital, Yonsei University College of Medicine. This study was approved by the institutional review board of Severance Hospital, Yonsei University (Seoul, Korea) and patients consents were waived.

### Data collection

Resting heart rate and blood pressure were checked at the time of 5-year survival upon the outpatient visit, and were measured in a quiet place with the patient in a sitting position with the feet flat on the floor after resting for 5–10 minutes. Before the test, eating, drinking alcohol, smoking, exercising, and bathing were avoided for 30 minutes. Baseline heart rate and blood pressure were measured by resting the arm on a table so that the cuff was at the same level as the heart.

To evaluate the severity of obesity, we measured weight, height, body mass index (BMI), and waist circumference, and used InBody 720 (Biospace, Korea) for body composition analysis. InBody 720 can automatically measure various parameters, intracellular and extracellular water, body fat mass, protein, and body cell mass. [16]. The normal standard range of body fat mass for males is defined at 10–20%, and 18–28% for females by Inbody 720. We followed the World Health Organization (WHO) Asia-Pacific classification to define the categories of obesity as normal (BMI < 23.0), overweight (23 ≤ BMI < 25), and obese (BMI ≥ 25) [17].

Physical activity data were collected through the interviewer-administered Global Physical Activity Questionnaire (GPAQ). GPAQ is a survey developed by the WHO to evaluate physical activity, and includes 16 questions involving activity at work, travel to and from places, recreational activity, and sedentary behavior [18]. We estimated total metabolic equivalent (MET)-hours/week from walking and vigorous exercise by summing the MET-hours/week from walking (hours per week multiplied by 3.5) and the MET-hours/week from vigorous exercise (hours per week multiplied by 7.0) [19,20].

Concerning colorectal adenoma characteristics, we analyzed the number, size, location, and pathology of colorectal polyps [21]. Any colorectal adenoma detected during surveillance colonoscopy was defined as recurrent adenoma. We defined advanced adenoma as a lesion of ≥ 10 mm, and a histologic finding of a villous component, or high-grade dysplasia [22]. Also, we reviewed about type of surgery, colorectal cancer stage, and receiving subsequent adjuvant chemotherapy.

### Statistical analyses

Means and standard deviations or medians and ranges were calculated for all continuous variables, as appropriate. Categorical variables were expressed as proportions (%) and statistical analyses were performed to compare the groups of variables. One-way analysis of variance (ANOVA) tests (or Kruskal-Wallis tests) were used to compare continuous variables, and chi-square tests (or Fisher’s exact test) were used for categorical variables, as appropriate. Kaplan-Meier analyses (log-rank tests) were carried out to compare the cumulative risk of advanced adenoma development across the quartiles of resting heart rates. Cox proportional hazards analyses were carried out to reveal the independent risk factors of the cumulative development of advanced adenoma with adjustment for various confounders, including age, sex, history of alcohol consumption, smoking, family history of CRC, DM, hypertension, skeletal muscle mass, body fat mass, physical activity, systolic blood pressure, diastolic blood pressure, resting heart rate, follow-up duration, time to surveillance colonoscopy, and number of surveillance colonoscopies. All statistical analyses were performed using the Statistical Package for Social Sciences (SPSS version 23.0; SPSS Inc., Armonk, NY, USA). A *p-*value of < 0.05 was considered statistically significant.

## Results

### Baseline characteristics

A total of 300 patients who were diagnosed with CRC and survived > 5 years after curative surgical resection were enrolled in this study. The CRC survivors were divided into 4 groups according to heart rate: quartile 1, 45–66 beat per minutes (b.p.m.); quartile 2, 67–73 b.p.m.; quartile 3, 74–90 b.p.m.; and quartile 4, 81–120 b.p.m.. There was no difference in education status, alcohol use, tobacco use, family history of CRC, height, weight, BMI, waist circumference, skeletal muscle mass, body cell mass, systolic blood pressure, diastolic blood pressure, tumor location, tumor stage, tumor grade, medical history, metabolic parameters, and medication use between the groups (**Table 1**). However, patients with low resting heart rate were older, had a higher percentage of men, undertook more physical activity, and had lower body fat mass, lower percentage body fat, and lower visceral fat area, although the difference in the average values was small.

**Table 1:** Baseline characteristics of colorectal cancer survivors according to resting heart rate.

Variables	Quartile 1 45–66 b.p.m. (n = 79)	Quartile 2 67–73 b.p.m. (n = 76)	Quartile 3 74–80 b.p.m. (n = 70)	Quartile 4 81–120 b.p.m. (n = 75)	*p value
**Age (years)**	62.4 ± 9.6	62.7 ± 10.2	59.9 ± 10.4	58.5 ± 12.0	0.041
**Males**	51 (64.6%)	38 (50.0%)	33 (47.1%)	36 (48.0%)	0.038
**Education**					0.477
**Less than high school**	16 (20.3%)	20 (26.3%)	18 (25.7%)	16 (21.3%)	
**High school graduate**	48 (60.8%)	41 (53.9%)	35 (50.0%)	39 (52.0%)	
**College degree or higher**	15 (19.0%)	15 (19.7%)	17 (24.3%)	20 (26.7%)	
**Current alcohol**	47 (59.5%)	37 (48.7%)	31 (44.3%)	38 (50.7%)	0.224
**Current tobacco**	37 (46.9%)	29 (38.1%)	29 (41.4%)	26 (34.6%)	0.210
**Family history of CRC**	9 (11.4%)	11 (14.5%)	8 (11.4%)	13 (17.3%)	0.395
**Physical activity (MET-hours/wk)**	29.5 ± 34.2	27.7 ± 34.2	18.3 ± 34.0	18.3 ± 2.7	0.049
**Height (cm)**	162.2 ± 7.3	162.8 ± 7.9	160.9 ± 7.8	161.7 ± 9.2	0.504
**Weight (kg)**	63.2 ± 9.7	65.1 ± 8.7	63.9 ± 10.3	64.7 ± 9.9	0.635
**BMI (kg/m**^**2**^**)**	24.0 ± 2.7	24.6 ± 3.1	24.7 ± 3.3	24.7 ± 3.1	0.363
**Waist (cm)**	86.0 ± 7.8	87.8 ± 8.2	86.6 ± 9.7	87.4 ± 8.3	0.596
**Skeletal muscle mass (kg)**	25.4 ± 5.2	25.1 ± 4.8	24.3 ± 4.8	24.8 ± 5.1	0.545
**Body Fat mass (kg)**	16.1 ± 5.3	18.9 ± 6.9	19.7 ± 6.8	19.5 ± 6.7	0.003
**Percent body fat (%)**	26.3 ± 7.8	28.6 ± 9.8	30.1 ± 8.0	29.8 ± 8.3	0.029
**Visceral fat area (cm**^**2**^**)**	74.0 ± 28.5	91.5 ± 40.6	94.3 ± 4.1	93.2 ± 38.0	0.003
**Body cell mass (kg)**	30.2 ± 5.7	29.8 ± 6.0	28.7 ± 5.3	29.1 ± 5.4	0.480
**Systolic blood pressure (mmHg)**	124.4 ± 14.4	127.5 ± 15.0	125.6 ± 15.6	125.3 ± 15.2	0.643
**Diastolic blood pressure (mmHg)**	77.2 ± 11.2	79.5 ± 11.5	78.7 ± 10.9	79.1 ± 9.8	0.554
**Tumor location**					0.436
**Colon**	46 (58.2%)	38 (50.0%)	37 (52.9%)	48 (64.0%)	
**Rectum**	33 (41.8%)	38 (50.0%)	33 (47.1%)	27 (36.0%)	
**Invasion through bowel wall**					0.056
**T1-2**	30 (38.0%)	41 (53.9%)	29 (41.4%)	20 (26.7%)	
**T3-4**	49 (62.0%)	35 (46.1%)	41 (58.6%)	55 (73.3%)	
**No. of positive lymph nodes**					0.896
**N1**	71 (89.9%)	71 (93.4%)	65 (92.9%)	68 (90.7%)	
**N2**	8 (10.1%)	5 (6.6%)	5 (7.1%)	7 (9.3%)	
**Stage of colorectal cancer**					0.087
**Stage 1**	28 (40.6%)	36 (46.1%)	27 (34.6%)	20 (26.7%)	
**Stage 2**	33 (47.8%)	35 (44.9%)	37 (47.4%)	48 (64.0%)	
**Stage 3**	8 (11.6%)	7 (9.0%)	14 (17.9%)	7 (9.3%)	
**Grade of differentiation**					0.345
**Well**	17 (21.5%)	26 (34.2%)	16 (22.9%)	18 (24.0%)	
**Moderate**	59 (74.7%)	48 (63.2%)	52 (74.2%)	51 (68.0%)	
**Poor/undifferentiated**	3 (3.8%)	2 (2.6%)	2 (2.9%)	6 (8.0%)	
**Type of operation**					0.517
**Low anterior resection**	31 (44.9%)	44 (56.4%)	40 (51.3%)	34 (45.3%)	
**Anterior resection**	14 (20.3%)	15 (19.2%)	19 (24.4%)	22 (29.3%)	
**Abdominopelvic resection**	0 (0.0%)	3 (3.8%)	4 (5.1%)	0 (0.0%)	
**Left hemicolectomy**	6 (8.7%)	2 (2.6%)	2 (2.6%)	3 (4.0%)	
**Right hemicolectomy**	16 (23.2%)	12 (15.4%)	13 (16.7%)	16 (21.3%)	
**Segmental resection**	2 (2.9%(	2 (2.6%)	0 (0.0%)	0 (0.0%)	
**Adjuvant chemotherapy**	7 (10.3%)	7 (9.0%)	13 (16.7%)	6 (8.0%)	0.954
**Medical history**					
**Hypertension**	23 (29.1%)	30 (39.5%)	25 (35.7%)	21 (28.0%)	0.784
**DM**	12 (15.2%)	10 (13.2%)	12 (17.1%)	17 (22.7%)	0.173
**Dyslipidemia**	15 (19.0%)	15 (19.7%)	12 (17.1%)	20 (26.7%)	0.322
**Coronary artery disease**	6 (7.6%)	3 (3.9%)	3 (4.3%)	0 (0.0%)	0.122
**Cerebrovascular disease**	0 (0.0%)	1 (1.3%)	4 (5.7%)	5 (6.7%)	0.058
**Peripheral arterial disease**	2 (2.5%)	0 (0.0%)	0 (0.0%)	1 (1.3%)	0.330
**Atrial fibrillation**	2 (2.5%)	1 (1.3%)	2 (2.9%)	2 (2.7%)	0.810
**Metabolic parameters**					
**Hemoglobin levels (g/dL)**	12 9 ± 1.8	13.3 ± 1.5	12.9 ± 1.8	12.9 ± 2.3	0.386
**Potassium (mmol/L)**	4.2 ± 0.3	4.2 ± 0.4	4.1 ± 0.4	4.1 ± 0.4	0.386
**Creatinine (mg/dL)**	0.9 ± 0.2	0.9 ± 0.2	0.9 ± 0.7	0.9 ± 0.2	0.501
**eGFR (ml/min/1.73m**^**2**^**)**	83.6 ± 15.2	87.8 ± 18.1	89.0 ± 22.0	88.8 ± 16.7	0.206
**Serum glucose (mg/dL)**	114.2 ± 61.9	107.0 ± 20.2	102.9 ± 22.3	108.4 ± 34.3	0.360
**Total cholesterol (mg/dL)**	175.3 ± 38.7	176.6 ± 43.6	179.8 ± 33.8	180.5 ± 33.9	0.803
**Medication**					
**Beta blocker**	6 (7.6%)	2 (2.6%)	2 (2.9%)	4 (5.3%)	0.420
**Diuretics**	1 (1.3%)	5 (6.6%)	2 (2.9%)	2 (2.7%)	0.298
**ACE inhibitor/ARB**	8 (10.1%)	9 (11.8%)	4 (5.7%)	8 (10.7%)	0.778
**Calcium channel blocker**	7 (8.9%)	5 (6.6%)	3 (4.3%)	4 (5.3%)	0.687
**Alpha blocker**	1 (1.3%)	1 (1.3%)	0 (0.0%)	1 (1.3%)	0.820
**Lipid-lowering medication**	21 (26.6%)	18 (23.7%)	17 (24.3%)	21 (28.0%)	0.924
**Aspirin**	8 (10.1%)	5 (6.6%)	9 (12.9%)	7 (9.3%)	0.643
**Clopidogrel**	4 (5.1%)	1 (1.3%)	6 (8.6%)	3 (4.0%)	0.220
**Anticoagulant**	1 (1.3%)	2 (2.6%)	0 (0.0%)	0 (0.0%)	0.312

Variables are expressed as mean ± SD or n (%).

*p value for comparing quartile groups based on resting heart rate.

b.p.m., beat per minute; CRC, colorectal cancer; METS, metabolic equivalents; BMI, body mass index; DM, diabetes mellitus; GFR, glomerular filtration rate; ACE, angiotensin converting enzyme; ARB, angiotensin II receptor antagonist; SD, standard deviation

### Colonoscopic surveillance

The mean follow-up period was 8.0 years (median, 7 years; range, 5–21 years). The colonoscopy was completely inserted into the cecum in all 300 patients. The mean duration between CRC surgery and polyp recurrence was 45.2 months (median, 48 months; range, 6–204 months). Sixty-one (20.3%) patients underwent 1 follow-up colonoscopy, 136 (45.3%) underwent 2, 86 (28.7%) underwent 3, and 17 (5.7%) underwent 4 or more follow-up colonoscopies. The number of follow-up colonoscopies was not significantly different among the 4 groups (*p* = 0.414). There was also no significant difference in the time to the first follow-up colonoscopy (16.9 months vs. 15.6 months vs. 18.7 months vs. 17.6 months, *p* = 0.635; **S1 Table**).

### Adenoma and advanced adenoma recurrence

A total of 162 (54.0%) patients showed 1 or more colorectal polyp. The total adenoma recurrence rate was 32.3%, and the total advanced adenoma recurrence rate was 9.0%. The tubular adenoma recurrence rate was 22.7% in the proximal colon and 14.3% in the distal colon and rectum. The advanced adenoma recurrence rate was 6.3% in the proximal colon and 3.3% in the distal colon and rectum.

### Resting heart rate and adenoma recurrence

The group with a low baseline heart rate showed a significantly lower recurrence rate of advanced adenoma than those with a high baseline heart rate (3.8% vs. 7.9% vs. 10.0% vs. 14.7%, *p* = 0.018; **Table 2**). There was no significant difference in the total adenoma recurrence rate.

**Table 2:** Colorectal polyp recurrence rates according to resting heart rates.

	Resting heart rate (b.p.m.)				*p* for trend
	Quartile 1 45–66 (n = 79)	Quartile 2 67–73 (n = 76)	Quartile 3 74–80 (n = 70)	Quartile 4 81–120 (n = 75)	
**Any polyp**					
**Event**	45 (57.0%)	39 (51.3%)	40 (57.1%)	38 (50.7%)	0.599
**Multivariable-adjusted**^**a**^	Referent	1.049 (0.648–1.697)	1.337 (0.812–2.201)	1.027 (0.625–1.688)	
**Hyperplastic polyp**					
**Event**	12 (15.2%)	13 (17.1%)	20 (28.6%)	15 (20.0%)	0.211
**Multivariable-adjusted**^**a**^	Referent	1.492 (0.614–3.625)	2.712* (1.144–6.427)	1.325 (0.541–3.236)	
**Tubular adenoma**					
**Event**	37 (46.8%)	32 (42.1%)	25 (35.7%)	27 (36.0%)	0.122
**Multivariable-adjusted**^**a**^	Referent	1.011 (0.564–1.810)	0.643 (0.322–1.287)	0.543 (0.274–1.077)	
**Advanced adenoma**					
**Event**	3 (3.8%)	6 (7.9%)	7 (10.0%)	11 (14.7%)	0.018
**Multivariable-adjusted**^**a**^	Referent	2.491 (0.431–14.394)	5.623* (0.981–32.241)	6.183** (1.181–32.373)	

Variables are expressed as n (%).

b.p.m., beat per minute

* *p* < 0.10

** *p* < 0.05

^a^ multivariate Cox proportional hazards analysis, including the confounders, such as age, sex, history of alcohol consumption, smoking, family history of CRC, DM, hypertension, skeletal muscle mass, body fat mass, physical activity, colorectal cancer stage, type of surgery, adjuvant chemotherapy, use of aspirin, systolic blood pressure, diastolic blood pressure, resting heart rate, follow-up duration, time to surveillance colonoscopy, and number of surveillance colonoscopies

### Physical activity, obesity, and resting heart rate

There was an association between vigorous exercise and heart rate (*p* for trend = 0.021); however, walking exercise and heart rate were not related (*p* for trend = 0.601; **S2 Table**). A correlation between total MET-hours/week and heart rate was also found (*p* for trend = 0.022), and the patient group with a low baseline heart rate showed a significantly higher proportion of active exercise (≥ 40 MET-hours/week) (27.8% vs. 30.3% vs. 11.4% vs. 13.3%, *p* for trend = 0.003). Furthermore, in the low heart rate group, more people undertook active exercise for more than 5 hours (44.3% vs. 41.3% vs. 28.6% vs. 29.3%, *p* for trend = 0.020). The mean heart rates of the active exercise group who had over 40 MET-hours/week and the sedentary group were 70.3 ± 10.5 and 75.0 ± 10.8 b.p.m. (*p* = 0.034), respectively.

Body fat mass and visceral fat area were also related to a higher heart rate (*p* for trend = 0.041 and 0.015, respectively); however, BMI and heart rate were not significantly associated (*p* for trend = 0.571; **S3 Table**). The mean heart rates of patients with a lower than average body fat mass (n = 10), with an average body fat mass (n = 104), and with a higher than average body fat mass (n = 186) were 65.4 ± 9.9, 73.4 ± 10.5, and 74.7 ± 10.9 b.p.m. (*p* = 0.024).

### Resting heart rate, physical activity, obesity, and adenoma recurrence

**Fig 1:**
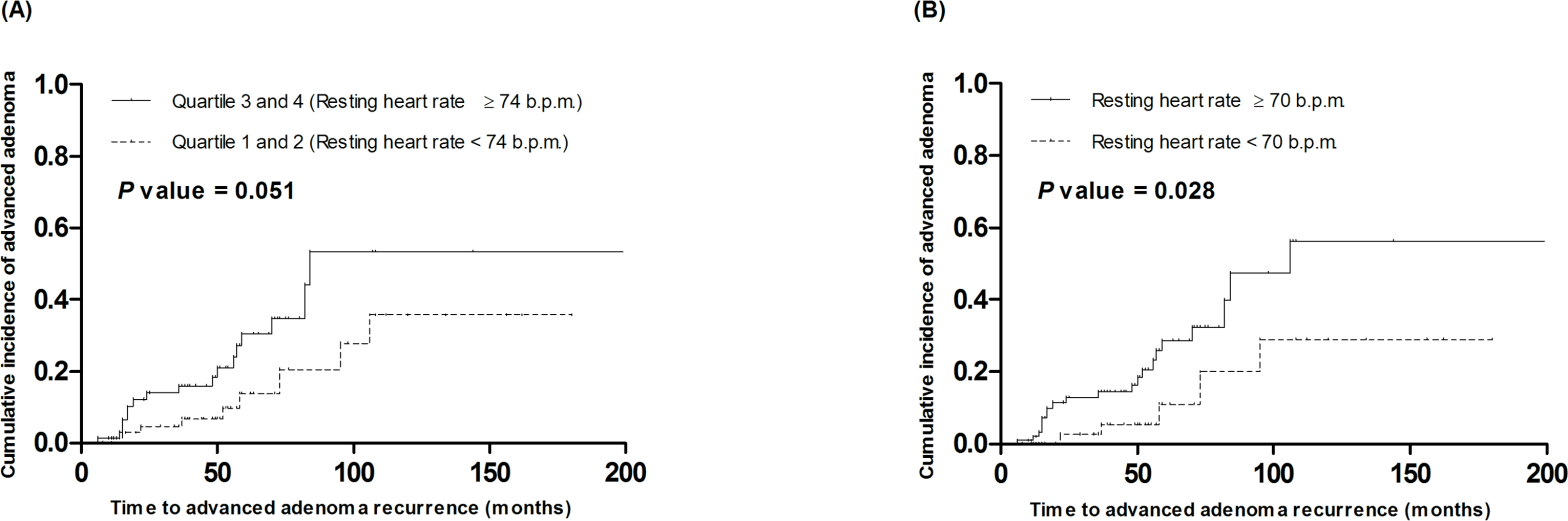
Kaplan-Meier analysis (log-rank tests) to compare the cumulative rate of development of advanced adenoma between low resting heart rate group (quartile 1 and 2, < 74 b.p.m.) and high resting heart rate group (quartile 3 and 4, ≥ 74 b.p.m.) (A), and between high resting heart rate group (≥ 70 b.p.m.) and low resting heart rate group (< 70 b.p.m.) (B).

We performed Kaplan-Meier analysis (log-rank tests) to compare the cumulative rate of the development of advanced adenoma between the low resting heart rate groups (quartile 1 and 2, < 74 b.p.m.) and high resting heart rate groups (quartile 3 and 4, ≥ 74 b.p.m.; **Fig 1A**). An increased resting heart rate was positively associated with the development of advanced adenoma, although it was not statistically significant (*p* = 0.051). However, a significant difference was found in the cumulative rate of advanced adenoma development between patients with a resting heart rate ≥ 70 b.p.m. and those with a rate < 70 b.p.m. (*p* = 0.028; **Fig 1B**). Subsequently, multivariate Cox proportional hazards analysis, including the confounders, such as age, sex, history of alcohol consumption, smoking, family history of CRC, DM, hypertension, skeletal muscle mass, body fat mass, physical activity, colorectal cancer stage, type of surgery, adjuvant chemotherapy, use of aspirin, systolic blood pressure, diastolic blood pressure, resting heart rate, follow-up duration, time to surveillance colonoscopy, and number of surveillance colonoscopies, showed that patients in the highest quartile of resting heart rate (≥ 81 b.p.m.) had a significantly higher risk of advanced adenoma recurrence (hazard ratio [HR]: 6.183, 95% confidence interval [CI]: 1.181–32.373, *p* = 0.031), compared to those in the lowest quartile (≤ 66 b.p.m.) (**Table 2**). Higher than normal body fat mass (hazard ratio [HR]: 10.743, 95% CI: 2.212–52.178, *p* = 0.003) and progressive colorectal cancer stage (stage 2 vs. stage 1; HR: 5.067, 95% CI: 1.446–17.756, *p* = 0.011) were also positively associated with increased risk of advanced adenoma recurrence (**Table 3**).

**Table 3:** Cox proportional hazards analysis for advanced adenoma recurrence.

Variables	Multivariate analysis		
	HR	95% CI	*p value
**Age (years)**	0.997	0.935–1.056	0.836
**Male sex**	1.736	0.340–8.874	0.508
**History of alcohol**	1.119	0.203–6.177	0.897
**History of smoking**	0.419	0.105–1.664	0.216
**Family history of colorectal cancer**	1.016	0.199–5.190	0.985
**Hypertension**	2.460	0.679–8.908	0.170
**DM**	0.656	0.148–2.895	0.577
**Skeletal muscle mass (kg)**	0.361	0.038–3.386	0.372
**Body Fat mass (kg)**	10.743	2.212–52.178	0.003
**Exercise amount (METs)**	1.006	0.988–1.025	0.492
**Colorectal cancer stage**			
**Stage 1**	1.000	(reference)	
**Stage 2**	5.067	1.446–17.756	0.011
**Stage 3**	0.139	0.000–152.492	0.581
**Type of surgery**	0.865	0.538–1.391	0.550
**Adjuvant chemotherapy**	2.669	0.002–2895.610	0.783
**Use of aspirin**	0.553	0.080–3.835	0.549
**Systolic blood pressure (mmHg)**	0.988	0.939–1.040	0.651
**Diastolic blood pressure (mmHg)**	1.026	0.956–1.102	0.469
**Heart rates**					
**Qualtile1**	1.000	(reference)	
**Qualtile2**	2.491	0.431–14.394	0.308
**Qualtile3**	5.623	0.981–32.241	0.053
**Qualtile4**	6.183	1.181–32.373	0.031
**Follow-up duration (years)**	0.891	0.732–1.085	0.252
**Time to first surveillence colonoscopy (months)**	0.975	0.947–1.004	0.094
**Number of surveillence colonoscopy**	1.094	0.400–2.993	0.864

HR, hazard ratio; CI, confidence interval; DM, diabetes mellitus

*p value for comparing advanced adenoma group and non-advanced adenoma group

**Table 4:** Subgroup analyses of multivariable-adjusted hazard ratios of advanced adenoma recurrence associated with heart rate.

	Resting heart rate (b.p.m.)				*p* for trend
	Quartile 1 45–66 (n = 79)	Quartile 2 67–73 (n = 76)	Quartile 3 74–80 (n = 70)	Quartile 4 81–120 (n = 75)	
**Body fat mass**					
Normal or less body fat mass (n = 114)					
Event	1 (2.9%)	0 (0.0%)	0 (0.0%)	1 (3.8%)	0.835
Multivariable-adjusted^a^	Referent	NS	NS	NS	
More than normal body fat mass (n = 186)					
Event	2 (4.5%)	6 (13.6%)	7 (14.3%)	10 (20.4%)	0.032
Multivariable-adjusted^a^	Referent	2.895 (0.461–18.174)	7.117** (1.142–44.351)	7.114** (1.133–44.686)	
**Physical activity**					
Active exercise (≥ 40 MET-hours/wk) (n = 63)					
Event	1 (4.5%)	2 (8.7%)	0 (0.0%)	0 (0.0%)	0.472
Multivariable-adjusted^a^	Referent	NS	NS	NS	
Sedentary (< 40 MET-hours/wk) (n = 237)					
Event	2 (3.5%)	4 (7.5%)	7 (11.3%)	11 (16.9%)	0.011
Multivariable-adjusted^a^	Referent	1.679 (0.267–10.563)	4.266* (0.820–22.204)	4.681* (0.923–23.725)	

Variables are expressed as n (%).

b.p.m., beat per minute

^a^Multivariable model adjusted for the same covariates used in Table 2.

* *p* < 0.10

** *p* < 0.05

In addition, we performed subgroup analysis according to the amount of exercise performed and body fat mass. In the higher than normal body fat mass group, 2 (4.5%), 6 (13.6%), 7 (14.3%), and 10 (20.4%) advanced adenomas were found in 44 patients with a resting heart rate of 45–66 b.p.m., in 44 patients with a resting heart rate of 67–73 b.p.m., in 49 patients with a resting heart rate of 74–80 b.p.m., and in 49 patients with a resting heart rate of 81–120 b.p.m., respectively (*p* = 0.032; **Table 4**). Furthermore, in the sedentary exercise group (< 40 MET-hours/week), 2 (3.5%), 4 (7.5%), 7 (11.3%), and 11 (16.9%) advanced adenomas were found in 57 patients with a resting heart rate of 45–66 b.p.m., in 53 patients with a resting heart rate of 67–73 b.p.m., in 62 patients with a resting heart rate of 74–80 b.p.m., and in 65 patients with a resting heart rate of 81–120 b.p.m., respectively (*p* = 0.011; **Table 4**). However, in patients with normal or less body fat mass or active exercise, resting heart rate was not a statistically significant independent factor for advanced adenoma recurrence. Therefore, the association of resting heart rate with advanced adenoma recurrence appeared to be stronger among patients who had more than normal body fat mass and sedentary life style.

## Discussion

A history of CRC is a very strong risk factor for the recurrence of advanced adenoma, and many studies have investigated the risk factors for polyp recurrence in patients with CRC [21,23]. Age, sex, a family history of CRC, and obesity were described as risk factors for colorectal neoplasia recurrence [24]. The study by Anker et al. reported that a high heart rate was an independent predictor of survival in cancer patients [10]. The study group was heterogeneous because it included various cancer types, such as colorectal (n = 36), pancreatic (n = 72), and non-small cell lung cancer (n = 37) (HR: 1.67, 95% CI: 1.01–2.78, *p* = 0.040). Van Kruiksdijk et al. showed that an elevated resting heart rate was related to the incident CRC (n = 67) risk in patients with manifest vascular disease (n = 6007) (HR: 1.19, 95% CI: 1.00–1.42) [11]. However, because of the high risk of cardiovascular mortality from vascular disease, the patients might not live long enough to develop cancer. With regard to colon polyps, there have been no data evaluated the association between resting heart rate and polyp recurrence.

In our study, surveillance colonoscopy after treatment of CRC revealed an adenoma recurrence rate of 32.3% and an advanced adenoma recurrence rate of 9.0%, which are similar to the results of previous studies showing an adenoma recurrence rate of 34.4–41.4% and an advanced adenoma recurrence rate of 4.4–6.5% [25–27]. With regard to the heart rate, multivariate analysis showed that an increased resting heart rate was associated with a significantly greater risk of advanced adenoma recurrence, but not with tubular adenoma and overall polyps. Our study showed a relationship between resting heart rate and advanced adenoma recurrence in the Cox proportional hazards analysis, with an HR of 11.550 (HR: 1.218–109.525, *p* = 0.033) for the highest quartile of resting heart rate compared with the lowest stratum. Furthermore, in subgroup analyses based on body fat mass and amount of exercise, our data showed that obese patients with a low resting heart rate had a lower risk of advanced adenoma than obese patients with a high resting heart rate. In addition, sedentary patients with a low resting heart rate had a valid lower risk of advanced adenoma than sedentary patients with a high resting heart rate. However, for patients with normal or less body fat mass or active exercise did not show any correlation between resting heart rate and advanced adenoma recurrence.

With regard to physical activity, our study demonstrated that a low heart rate was positively associated with exercise for more than 5 hours or over 40 MET-hours/week. If aerobic exercise is performed for a long time, it affects the parasympathetic nerve, thus increasing the stroke volume and lowering the resting heart rate [28]. It is well known that bradycardia syndrome (< 60 b.p.m.) occurs in athletes, especially in those who undertake marathon running [29]. In terms of obesity, our data showed that a low heart rate was negatively associated with body fat mass and the visceral fat area, but not with BMI. We already showed usefulness of body composition analysis and its relationship with colorectal polyp recurrence [30]. BMI may be an inaccurate measure of percentage body fat for an individual [31]. For example, greater loss of muscle mass with age exacerbates the prevalence of false-negative BMIs. Meanwhile, our results also showed that low body fat mass and more exercise could contribute to lowering the rate of advanced adenoma recurrence associated with resting heart rate.

As mentioned above, active exercise was directly related to low body fat mass and low resting heart rate. However, in multivariate analysis and Cox proportional hazards analysis after adjustment for related factors, including these 3 factors, resting heart rate was still remained as a significant independent factor associated with a lower rate of advanced adenoma recurrence. Therefore, we need to understand the mechanism of the tumor preventive effect of a lower resting heart rate, which is not related to the direct effects of active exercise. In this regard, the molecular biologic mechanism of the relationship between heart rate and tumor development has not yet been clearly demonstrated. In a previous study, the catecholamine stress hormone norepinephrine was shown to influence tumor progression by modulating the expression of factors implicated in angiogenesis and metastasis [32]. Masur et al. reported that norepinephrine-induced locomotion of SW 480 colon carcinoma cells is mediated by the β2-adrenoceptor and inhibited by beta blockers [33]. Yang et al. suggested that norepinephrine could stimulate the aggressive potential of melanoma cancer cells by inducing the production of vascular endothelial growth factor (VEGF), interleukin (IL)-8, and IL-6 [34]. In addition, an epidemiological meta-analysis demonstrated that beta blocker use is associated with improved overall survival (HR: 0.79, 95% CI: 0.67–0.93, *p* = 0.004) and disease free survival (HR: 0.69, 95% CI: 0.53–0.91, *p* = 0.009) in cancer patients [35]. Recently, Choi et al. reported that perioperative propranolol was effective in reducing the ovarian cancer burden measured using CA 125, suggesting its potential benefits in decreasing perioperative tumor growth (*p* = 0.044) [36]. Therefore, an intervention targeting components of the activated sympathetic-adrenal medullary axis or the utilization of beta blockers might represent new strategies for slowing the progression of malignant disease and improving cancer patients’ quality of life. Furthermore, this new strategy might be applicable to cancer prevention in the future.

This study has the innate limitations of a retrospective cross-sectional case-control study performed in a single-tertiary university hospital. Another weak point was that only a single measurement of physical activity, body composition data, and resting heart rate at least 5 years after the diagnosis and treatment of CRC was available. The serial measurements during 5 years would provide more useful information. However, our study has the strong points of including a patient group with a history of CRC that is a high risk group of colorectal neoplasia, having detailed long-term follow-up surveillance data, and analyzing detailed exercise amount and body composition, which are related with resting heart rate. In addition, because 5-year survivors of CRC were included, and physical activity, body composition, and resting heart rate were measured after a minimum period of 5 years from the initial diagnosis and treatment of CRC, these data may be reflective of a stabilized status without variance from the impact of cancer treatment or cancer-related stress. Last weak point was that we had relative small sample size and few recurrence events. Further studies with larger sample size, longer follow-up and more events will be needed.

In conclusion, we found that a high resting heart rate were meaningful independent risk factors of advanced adenoma recurrence in CRC survivors.

## Supporting information

S1 Table Comparison of total number and interval of follow-up colonoscopies among quartile heart rate groups. (PDF)

S2 Table Resting heart rates according to exercise type and amounts. (PDF)

S3 Table Resting heart rates according to obesity index. (PDF)
