# Supplementary material for: Resting heart rate is an independent predictor of advanced colorectal adenoma recurrence
Source: PLoS One. 2018 Mar 2;13(3):e0193753. doi: 10.1371/journal.pone.0193753 (PMC5834177; doi:10.1371/journal.pone.0193753)
Supplement: S1 Table — (PDF) [file pone.0193753.s001.pdf]

S1 Table.

**Variables**	**Quartile 1 45-66 b.p.m. (n=79)**	**Quartile 2 67-73 b.p.m. (n=76)**	**Quartile 3 74-80 b.p.m. (n=70)**	**Quartile 4 81-120 b.p.m. (n=75)**	***p* value**
**Number of follow-up colonoscopies**					0.414
1	16 (20.3%)	13 (17.1%)	16 (22.9%)	17 (22.7%)	
2	44 (55.7%)	36 (47.4%)	26 (37.1%)	30 (40.0%)	
3	14 (17.7%)	24 (31.6%)	24 (34.3%)	24 (32.0%)	
≥4	5 (6.3%)	3 (3.9%)	4 (5.7%)	4 (5.3%)	
**Interval to first follow-up colonoscopy (months)**					0.635
	16.9 ± 14.7	15.6 ± 11.1	18.7 ± 16.3	17.6 ± 15.7	

Variables are expressed as mean ± SD or n (%).

**p* value for comparing quartile groups based on resting heart rate.

b.p.m., beat per minute; SD, standard deviation
