# Supplementary material for: Resting heart rate is an independent predictor of advanced colorectal adenoma recurrence
Source: PLoS One. 2018 Mar 2;13(3):e0193753. doi: 10.1371/journal.pone.0193753 (PMC5834177; doi:10.1371/journal.pone.0193753)
Supplement: S2 Table — (PDF) [file pone.0193753.s002.pdf]

**S2 Table.**

| | Quartile 1<br>45-66 b.p.m.<br>(n=79) | Quartile 2<br>67-73 b.p.m.<br>(n=76) | Quartile 3<br>74-80 b.p.m.<br>(n=70) | Quartile 4<br>81-120 b.p.m.<br>(n=75) | *p* trend |
|---|---|---|---|---|---|
| **Vigorous exercise (hour/wk)** | | | | | 0.021 |
| none | 44 (55.7%) | 41 (53.9%) | 46 (65.7%) | 50 (66.7%) | |
| 0–1hours | 1 (1.3%) | 0 (0.0%) | 3 (4.3%) | 0 (0.0%) | |
| 1–2hours | 6 (7.6%) | 13 (17.1%) | 14 (20.0%) | 7 (9.3%) | |
| 3–4hours | 9 (11.4%) | 4 (5.3%) | 1 (1.4%) | 7 (9.3%) | |
| 5–6hours | 6 (7.6%) | 4 (5.3%) | 2 (2.9%) | 4 (5.3%) | |
| ≥7hours | 13 (16.5%) | 14 (18.4%) | 4 (5.7%) | 7 (9.3%) | |
| **Walking exercise (hour/wk)** | | | | | 0.601 |
| none | 45 (57.0%) | 42 (55.3%) | 41 (58.6%) | 43 (57.3%) | |
| 0-1hours | 1 (1.3%) | 0 (0.0%) | 0 (0.0%) | 0 (0.0%) | |
| 1-2hours | 7 (8.9%) | 15 (19.7%) | 8 (11.4%) | 8 (10.7%) | |
| 3-4hours | 9 (11.4%) | 5 (6.6%) | 10 (14.3%) | 13 (17.3%) | |
| 5-6hours | 3 (3.8%) | 6 (7.9%) | 5 (7.1%) | 3 (4.0%) | |
| ≥7hours | 14 (17.7%) | 8 (10.5%) | 6 (8.6%) | 8 (10.7%) | |
| **Walking and vigorous exercise (MET-hours/wk)** | | | | | 0.022 |
| none | 23 (29.1%) | 18 (23.7%) | 27 (38.6%) | 28 (37.3%) | |
| 0-5METs | 2 (2.5%) | 3 (3.9%) | 1 (1.4%) | 1 (1.3%) | |
| 5-9METs | 3 (3.8%) | 9 (11.8%) | 6 (8.6%) | 5 (6.7%) | |
| 10-19METs | 11 (13.9%) | 14 (18.4%) | 16 (22.9%) | 14 (18.7%) | |
| 20-39METs | 18 (22.8%) | 9 (11.8%) | 12 (17.1%) | 17 (22.7%) | |
| ≥40METs | 22 (27.8%) | 23 (30.3%) | 8 (11.4%) | 10 (13.3%) | |
| **Active exercise group (≥ 40 MET-hours/week)** | 22 (27.8%) | 23 (30.3%) | 8 (11.4%) | 10 (13.3%) | 0.003 |
| **Active exercise group (≥ 5hours/week)** | 35 (44.3%) | 31 (41.3%) | 20 (28.6%) | 22 (29.3%) | 0.020 |

b.p.m., beat per minute; MET, metabolic equivalent
