# Supplementary material for: Resting heart rate is an independent predictor of advanced colorectal adenoma recurrence
Source: PLoS One. 2018 Mar 2;13(3):e0193753. doi: 10.1371/journal.pone.0193753 (PMC5834177; doi:10.1371/journal.pone.0193753)
Supplement: S3 Table — (PDF) [file pone.0193753.s003.pdf]

S3 Table.

	**Quartile 1 45-66 b.p.m. (n=79)**	**Quartile 2 67-73 b.p.m. (n=76)**	**Quartile 3 74-80 b.p.m. (n=70)**	**Quartile 4 81-120 b.p.m. (n=75)**	***p* trend**
**BMI**					0.571
Normal (BMI < 23) (n=94)	27 (34.2%)	21 (27.6%)	22 (31.4%)	24 (32.0%)	
Overweight (23≤ BMI < 25) (n=83)	24 (30.4%)	21 (27.6%)	20 (28.6%)	18 (24.0%)	
Obese (BMI ≥ 25) (n=123)	28 (35.4%)	34 (44.7%)	28 (40.0%)	33 (44.0%)	
**Body fat mass**					0.041
Lower than average (n=10)	5 (50.0%)	3 (30.0%)	1 (10.0%)	1 (10.0%)	
Average (n=104)	30 (28.8%)	29 (27.9%)	20 (19.2%)	25 (24.0%)	
Higher than average (n=186)	44 (23.7%)	44 (23.7%)	49 (26.3%)	49 (26.3%)	
**Visceral fat area**					0.015
Lower than average (n=36)	13 (36.1%)	7 (19.4%)	5 (13.9%)	11 (30.6%)	
Average (n=176)	55 (31.3%)	45 (25.6%)	38 (21.6%)	38 (21.6%)	
Higher than average (n=88)	11 (12.5%)	24 (27.3%)	27 (30.7%)	26 (29.5%)	

b.p.m., beat per minute; BMI, body mass index
